# Exercise-Induced Anaphylaxis: A Case Report and Review of the Diagnosis and Treatment of a Rare but Potentially Life-Threatening Syndrome

**DOI:** 10.1155/2013/610726

**Published:** 2013-03-27

**Authors:** Nathan T. Jaqua, Matthew R. Peterson, Karla L. Davis

**Affiliations:** ^1^Department of Internal Medicine, Tripler Army Medical Center, 1 Jarrett White Road, Honolulu, HI 96859, USA; ^2^Department of Allergy and Immunology, Tripler Army Medical Center, 1 Jarrett White Road, Honolulu, HI 96859, USA

## Abstract

A 24-year-old male Marine with an uncomplicated medical history and a long history of strenuous, daily exercise presented to the emergency department after experiencing anaphylactic shock while running. Symptoms resolved following administration of intramuscular diphenhydramine, ranitidine, intravenous methylprednisolone, and intravenous fluids. On followup in the allergy clinic, a meticulous clinical history was obtained which elucidated a picture consistent with exercise-induced anaphylaxis. He had experienced diffuse pruritus and urticaria while exercising on multiple occasions over the last three years. His symptoms would usually increase as exercise continued. Prior to the first episode, he regularly exercised without symptoms. Exercise-induced anaphylaxis is a rare but potentially life-threatening syndrome that requires a careful clinical history and is a diagnosis of exclusion. Treatment is primarily exercise avoidance. Prophylactic mediations are inconsistently effective but are empirically used. Successful treatment with omalizumab was recently reported in a case of refractory exercise-induced anaphylaxis.

## 1. Introduction

Exercise-induced anaphylaxis (EIAn) is a rare but potentially life-threatening syndrome. It presents with signs and symptoms that occur during or shortly following exercise. Signs and symptoms include sudden fatigue, warmth, pruritus, flushing, and urticarial and may progress to anaphylactic shock including upper airway compromise secondary to bronchospasm or circulatory collapse. A strong clinical suspicion, exclusion of other potential diagnoses, and a meticulous clinical history are required for diagnosis. The history includes character and intensity or exercise, as well as environmental variables, and it may also elucidate triggers including temperature or medications. Treatment is primarily avoidance, never exercising alone, and always carrying an epinephrine autoinjector. 

## 2. Case Report

A 24-year-old-male presented to the emergency department (ED) via ambulance for symptomatic hypotension he experienced while running. Approximately ten minutes into his run, he began to experience generalized pruritis, diffuse urticaria, palpitations, and lightheadedness. He sat to rest for several minutes and when he attempted to stand he became more lightheaded and felt like he was going to “black out.” However, he did not lose consciousness. Evaluation by EMS in the field revealed a blood pressure (BP) of 60s over palpable. He received one liter of normal saline (NS) en route to the ED along with 0.3 mg intramuscular epinephrine and 50 mg of intramuscular diphenhydramine. His BP upon arrival to the ED was 113/69 with a heart rate of 87, a respiratory rate of 20, a temperature of 98.1 F oral, and with SpO2 of 98% on room air. He had no wheezing or dyspnea.

In the ED, he received one additional liter of NS along with 125 mg intravenous methylprednisolone and 50 mg intravenous ranitidine. He remained stable in the ED, and upon discharge his BP was 124/67, which is near his baseline. He was advised to avoid exercise and was discharged with an epinephrine autoinjector, fexofenadine at 60 mg daily, oral prednisone 60 mg daily for five days, and a referral to allergy for an outpatient evaluation.

In the allergy clinic, the patient reported a 3-year history of diffuse pruritus followed by diffuse urticaria during or shortly after running. He experienced approximately 20–25 episodes over the last three years. He had been evaluated in the acute care clinic multiple times and treated with oral or intramuscular diphenhydramine with symptom resolution. On four occasions, he experienced anaphylaxis in addition to urticaria and pruritus. The most recent and serious of which is described earlier occurring three weeks prior to allergy evaluation. Symptoms have not consistently been associated with foods, medications, or environmental conditions. He stated he usually runs in the morning on an empty stomach. He had not had any urticaria or pruritus while weight training; he only experiences symptoms while running. He had minimal benefit from prophylactic H1 antihistamines. 

Given the unpredictable and potentially lethal nature of EIAn, he was advised to avoid running, carry an epinephrine autoinjector at all times, and exercise with a partner trained in symptom recognition and administration of the epinephrine autoinjector. He was also instructed to avoid food, NSAIDs, and alcohol a minimum of four hours prior to exercise. He was advised to engage in a graded exercise program and at the first indication of flushing, itching, hives, lightheadedness, or shortness of breath to cease exercising, self-administer epinephrine, and seek immediate medical assistance. 

## 3. Discussion

Anaphylaxis is a life-threatening, multisystem syndrome caused by the sudden release of basophil and mast cell mediators. It is often underdiagnosed likely due to commonly presenting without overt shock or obvious allergic signs or symptoms (e.g., skin signs). Anaphylaxis ranges from mild signs and symptoms to anaphylactic shock. Rapid identification by emergency personnel is important so that treatment can be started prior to progression to respiratory or circulatory collapse. A consensus definition based on clinical criteria for diagnosing anaphylaxis was outlined in 2006 by a panel of multidisciplinary experts. The panel defined a clear set of criteria which they proposed would have a sensitivity of 95% [1]. 

Per the criteria, anaphylaxis is highly likely when one of three criteria is fulfilled. The first criterion includes the acute onset of illness with involvement of skin and/or mucosa (e.g., hives, pruritus, and angioedema) and either respiratory compromise or a systolic BP < 90 mmHg (or symptomatic hypotension). Another criterion consists of two or more signs or symptoms rapidly occurring following exposure to a likely allergen. The third criterion is defined as systolic BP < 90 mmHg or symptomatic hypotension following exposure to a known allergen [1]. 

The most frequent triggers of anaphylaxis include food, insect stings, and medications; however, nonimmunologic mechanisms may also provoke anaphylaxis. Other known triggers include anesthetics, latex, and seminal fluid. Food tends to be more frequently implicated in children, whereas medications and insect stings are more common causes of anaphylaxis in adults. Previously, anaphylaxis was considered to be IgE-mediated and non-IgE-mediated reactions were referred to as anaphylactoid. In October of 2003, the World Allergy Organization (WAO) proposed revised nomenclature criteria [2]. The term allergic anaphylaxis now refers to a reaction that is mediated via an immunologic mechanism. These mechanisms include IgE and IgG or immune complex complement related. IgE antibody reactions may also be more specifically characterized as “IgE-mediated allergic anaphylaxis.” Conversely, nonimmunologic etiologies should be referred to as nonallergic anaphylaxis. 

EIAn presents with signs and symptoms occurring during or shortly after exercising. Typical signs and symptoms include fatigue, warmth, pruritus, flushing, and urticaria. In extreme cases, it may progress to wheezing, angioedema, airway compromise, or circulatory collapse. Other symptoms may include nausea, cramping, or diarrhea [3–5]. Generalized pruritus and urticaria are often the initial manifestation and the urticaria is usually ≥10 mm in diameter [5]. Bronchospasm has been reported; however, the incidence is considered to be lower than in other etiologies of anaphylaxis [4]. Many patients engage in regular exercise without consistent reproducibility of signs or symptoms; however, some experience a single episode of anaphylaxis over years of exercise and others experience monthly or even more frequent episodes [5]. Most patients exercise for years before experiencing their first episode. 

Along with EIAn, there is also food-dependent exercise-induced anaphylaxis (FDEIAn). There are known triggers including NSAIDs, alcohol, high heat or humidity, and cold exposure [6, 7]. However, the most commonly reported trigger is food ingestion prior to exercise [6]. Trigger foods that have been reported include Crustacea (shrimp and crab), wheat, grains, nuts, fruits, vegetables, legumes, and seeds. Less commonly implicated foods are meats, eggs, and cow's milk [8–13]. Interestingly, there is a case reported in an individual with food-dependent exercise-induced anaphylaxis (FDEIAn) provoked by ingestion of tofu prior to exercise; however, ingestion of soy milk had no such effect, suggesting the significance of food processing. 

EIAn and FDEIAn are considered rare. The previously reported frequency of FEIAn was 0.21% of junior-high-school students among a total of 3,753 [14]. However, another more recent epidemiological study involved 76,229 junior-high-school students in Japan. This study found that the frequency of EIAn and FEIAn was 0.031% and 0.017%, respectively. They also found no difference between sexes [15]. 

The pathophysiology of EIAn and FDEIAn is not completely understood; however, mast cell degranulation with subsequent histamine release is thought to play a primary role. Although the specific events responsible for mast cell activation are unknown, an association between mast cell degranulation, vasoactive mediators (including elevated plasma histamine), and EIAn exists [5, 16–18]. Other proposed potential mechanisms include exercise-induced changes in blood flow, antigen presentation, blood pH, and gut permeability [16, 18]. Gastrin is among the mast cell secretagogues known to be released during exercise [19]. 

EIAn is a diagnosis of exclusion. A meticulous clinical history should be obtained and other potential diagnoses should be excluded. The differential includes cold-induced urticaria and anaphylaxis, exercise-induced asthma, cholinergic urticaria, mastocytosis, and FDEIAn. 

FDEIAn is also suggested by clinical history in the context of exercise following ingestion of food. A careful history may elucidate the culprit food. Like in EIAn, the diagnosis is one of exclusion. In contrast to EIAn, if there appears to be a specific food trigger, skin testing or IgE immunoassays may be helpful, as treatment involves specific food avoidance. Alternatively, selective reintroduction of foods into the diet may clarify the provoking food. 

In both EIAn and FDEIAn, exercise challenge testing is not required for diagnosis. Given the lack of validated exercise protocols and the lack of reproducibility of symptoms, exercise testing is often not performed [3]. 

Treatment of EIAn and FDEIAn is preventative, largely by avoidance, as there are no randomized controlled trials of therapy. In FDEIAn, if a specific trigger food is identified, avoidance of the food for four to six hours prior to exercise is usually effective in preventing attacks. Other identified triggers such as NSAIDs, high humidity or heat, and alcohol should also be avoided. 

Prophylactic pharmacotherapy has not been systematically studied; however, there are published case reports and rationalized empiric treatments. Although the effect of H1 antihistamines is inconsistent, administration prior to strenuous exercise may reduce symptoms; however, it is usually not completely effective. Beta agonists and phosphodiesterase-inhibiting medications have not demonstrated any benefit in preventing attacks [3]. Other therapies reported but not systematically studied include leukotriene inhibitors, misoprostol, and oral corticosteroids [20, 21]. 

One recent case report details the successful treatment of EIAn with omalizumab, an immunoglobulin E (IgE) monoclonal antibody [22]. Omalizumab is approved for treatment of moderate to severe allergic asthma. Successful off-label therapy with omalizumab for a variety of other conditions has been reported to include mastocytosis, venom induced, and idiopathic anaphylaxis [18, 22–26]. However, it had not been previously reported in the treatment of EIAn. As omalizumab does not interact with all proposed pathways involved in EIAn, it is hypothesized the mast cell stabilizing effect is the predominant therapeutic mechanism [22]. 

## 4. Conclusion

EIAn is a rare but potentially life-threatening syndrome presenting with signs and symptoms of anaphylaxis during or shortly after exercise. It is a clinical diagnosis of exclusion confirmed by a meticulous history. Treatment is primarily avoidance of identified triggers, exercising with a partner trained in sign and symptom recognition as well as administration of an epinephrine autoinjector, and prophylactic pharmacotherapy for potential reduction in severity. If the patient presents with signs or symptoms of anaphylaxis or anaphylactic shock, treatment is the same as conventional therapy (e.g., epinephrine, etc). A graded exercise program should be undertaken to ascertain a safe level of exercise. EIAn is unpredictable and extreme caution should always be advised.
